# The Importance of Stromal Endometriosis in Thoracic Endometriosis

**DOI:** 10.3390/cells10010180

**Published:** 2021-01-18

**Authors:** Ezekiel Mecha, Roselydiah Makunja, Jane B. Maoga, Agnes N. Mwaura, Muhammad A. Riaz, Charles O. A. Omwandho, Ivo Meinhold-Heerlein, Lutz Konrad

**Affiliations:** 1 Department of Biochemistry, University of Nairobi, Nairobi 00100, Kenya; ezekiel_mecha@yahoo.com (E.M.); lydiarozy@yahoo.com (R.M.); charlesomwandho@gmail.com (C.O.A.O.); 2 Institute of Gynecology and Obstetrics, Faculty of Medicine, Justus Liebig University, 35392 Giessen, Germany; janeykemunto@gmail.com (J.B.M.); njokimwaura8@gmail.com (A.N.M.); assad_riaz2k@yahoo.com (M.A.R.); ivo.meinhold-heerlein@gyn.med.uni-giessen.de (I.M.-H.); 3 Deputy Vice Chancellor, Kirinyaga University, Kerugoya 10300, Kenya

**Keywords:** endometrium, stromal endometriosis, thoracic endometriosis, Sampson, pathogenesis, endometriosis

## Abstract

Thoracic endometriosis (TE) is a rare type of endometriosis, where endometrial tissue is found in or around the lungs and is frequent among extra-pelvic endometriosis patients. Catamenial pneumothorax (CP) is the most common form of TE and is characterized by recurrent lung collapses around menstruation. In addition to histology, immunohistochemical evaluation of endometrial implants is used more frequently. In this review, we compared immunohistochemical (CPE) with histological (CPH) characterizations of TE/CP and reevaluated arguments in favor of the implantation theory of Sampson. A summary since the first immunohistochemical description in 1998 until 2019 is provided. The emphasis was on classification of endometrial implants into glands, stroma, and both together. The most remarkable finding is the very high percentage of stromal endometriosis of 52.7% (CPE) compared to 10.2% (CPH). Chest pain, dyspnea, right-sided preference, and diaphragmatic endometrial implants showed the highest percentages in both groups. No significant association was found between the recurrence rate and the various appearances of endometriosis. Sometimes in CPE (6.8%) and CPH (30.6%) no endometrial implants were identified underlining the importance of sensitive detection of endometriosis during and after surgery. We suggest that immunohistochemical evaluation should become mandatory and will improve diagnosis and classification of the disease.

## 1. Endometriosis

Endometriosis is characterized by implantation and growth of endometrial glands and stroma outside the uterine cavity [1]. Of note, endometriotic glands almost always have an overtly endometrial appearance and histologically resemble uterine endometrial glands [1]. Nonetheless, endometriotic lesions show many variations in color, depth of invasion, adhesions, ovarian cysts, and different epithelial to stromal cell ratios up to the extreme case of stromal endometriosis [2]. Although pathogenesis of endometriosis is still debated, retrograde menstruation followed by implantation of the endometrial tissue on different surfaces [3] in pelvic or extra-pelvic locations is generally accepted as the main cause of endometriosis. Additionally, alternative hypotheses such as the coelomic metaplasia theory [4], the genetic/epigenetic theory [5], circulating stem/progenitor cells [6], repeated tissue injury and repair (ReTIAR) caused by uterine hyperperistalsis [7], and a fetal [8] or adolescent [9] origin have been also suggested.

The greatest enigma in pathogenesis of endometriosis is still the unanswered question of why, despite a high rate of retrograde menstruation, only approximately 1–10% of women in their reproductive age acquire endometriosis [10]. This clearly indicates the importance of additional causative factors like inflammation, oxidative stress [11], disturbance of the peritoneal barrier [12], and genetic/epigenetic changes [5], which then affect migration, adhesion, and invasion of endometrial cells at ectopic sites.

Besides elucidation of these risk factors that favor initiation of endometriosis, the question whether or not the endometrium is the main if not the only source for ectopic endometrial cells needs to be answered as it impacts also strongly the therapy of the disease.

Therapeutically, the decision whether it is sufficient to eliminate only the ectopic implant(s) or whether endometrium or uterus must also be examined carefully, is crucial. As clearly shown, the overall recurrence rates were lowest for patients who underwent hysterectomy together with laparoscopy compared to laparoscopy alone [13]. These data clearly indicate that the endometrium is the main source for ectopic endometrial cells, although this does not exclude other hypotheses regarding the pathogenesis of endometriosis.

Since many arguments have been put forward to criticize the implantation hypothesis such as the occurrence of extra-pelvic endometriosis [14], the thoracic endometriosis syndrome is discussed in the following chapters with a special focus on catamenial pneumothorax (CP) as one of the most common symptoms of thoracic endometriosis (TE).

## 2. Thoracic Endometriosis Syndrome

Thoracic endometriosis as characterized by endometrial implants in or around the lungs is classified into CP, catamenial hemothorax, catamenial hemoptysis, catamenial hemopneumothorax, and endometriosis in lung nodules and few other presentations [14]. Deposition of endometrial implants was observed on the diaphragm (78.82%), on the pleura (14.33%), the lungs (4.46%) and in all three locations (1.11%) as summarized in a recent review with *n* = 628 TE patients [15]. It has been proposed that histological findings of TE in patients with pneumothorax should be termed thoracic endometriosis-related pneumothorax (TERP), regardless of whether the onset of pneumothorax met the criteria for CP or not [16]. However, we refer in this review to CP, because this terminology has been preferentially used in most manuscripts. CP is clinically defined as a recurrent pneumothorax of at least two episodes occurring within 24 h before to 72 h after the onset of menstruation in women of reproductive age [17].

### 2.1. Epidemiology

Some large scale studies with patients demonstrated that CP accounted for 3.15% (873/27,716) [18], 0.90% (6/664) [19] and 5.61% (11/196) [20] resulting in 3.11% of CP cases in the group with pneumothorax. In France the incidence of spontaneous primary pneumothorax in women was estimated to be 6.93/100,000 (*n* = 9963) [21]. Additionally, in a recent study conducted in Japan with a very high number of CP cases (*n* = 27,716) the mean age was found to be 37.9 ± 7.7 [18].

### 2.2. Symptoms and Diagnosis

Thoracic endometriosis is characterized mainly by clinical presentations such as; CP (72%), catamenial hemothorax (13%), catamenial hemoptysis (10%), and lung nodules (4%) [22]. The most common CP symptoms are chest/shoulder pain, dyspnea (shortness of breath), and cough [22]. The right-sided location was reported to be 80% in a recent review [15].

Diagnosis of TE is often greatly delayed leading to further complications of the disease and recurrent hospitalizations. Most often chest X-ray (CTX) (31%) and chest computed tomography (CT) (14.3%) are used (reviewed in [15]). Contrary, in only one study MRI was investigated for the detection of diaphragmatic endometriosis despite a high sensitivity of 83% [23]. Notably, the accuracy of imaging methods for the detection of TE has not been evaluated in depth.

In two studies, predictors of CP have been presented: age > 25 years (especially 36–45 years), coexisting endometriosis, right-sided pneumothorax, history of hospitalization, lower probability for over- or underweight and ex-smoker or current smoker [18]. Similarly, Haga et al. [16] identified the following significant parameters: right-sided pneumothorax, history of pelvic endometriosis, age ≥ 31 years, and no smoking history. These factors were assigned 6, 5, 4, and 3 points, respectively, to establish a scoring system from 0 to 18. Cutoff values ≥ 12 yielded the highest positive predictive value (86%) and negative predictive value (95.2%) for the prediction of primary spontaneous pneumothorax. However, up to date, the scoring was never evaluated prospectively.

### 2.3. Correlation of TE with Pelvic Endometriosis

A recent review reported concomitant appearance of TE with pelvic endometriosis at 52.9% [15]. On the other side, a previous study (*n* = 3008) of cases with pelvic endometriosis revealed only 46 cases (1.53%) with diaphragmatic lesions [24]. Thus, pelvic endometriosis is a risk factor or an indicator of susceptibility, but not a prerequisite for TE.

### 2.4. Histological and Immunohistochemical Characterization of TE

In 2007 Alifano et al. [25] stated that only the appearance of ectopic endometrial stroma together with glands can prove TE and that if only stroma is present diagnosis of TE is probable. This is equivalent to the definition of pelvic endometriosis [1]. To the best of our knowledge, the first immunohistochemical study of TE was presented by Flieder et al. in 1998 [26], who reported the identification and characterization of four cases with ectopic endometrial glands/stroma in the pleura with antibodies discriminating between epithelial, stromal, vascular, and neuroendocrine tissue.

Sensitivity of CD10 for the detection of the stromal compartment in TE was tested; and was as high as 88.1% (74/84) [27] and 96.8% (60/62) [28]. Furthermore, in 54.8% (46/84) of cases metaplasia of ectopic endometrial cells to smooth muscle cells was observed [27], a phenomenon also reported for pelvic endometriosis [2].

Except for CP, lung resections for various types of pneumothorax demonstrated negative staining (0/50) with CD10 and hormonal receptors, estrogen receptor (ER) and progesterone receptor (PR) [29]. Interestingly, stromal cells in the lung with a scattered pattern were negative for CD10, whereas stromal cells with an aggregated pattern expressed CD10 and thus proved to be endometrial implants [30].

Detection of ectopic endometrial epithelial and stromal cells in TE was most often performed with ER, PR, and CD10, but recently interferon-inducible transmembrane protein 1 (IFITM1) for stromal [28] and Pax8 for epithelial cells [31] seem to be also highly sensitive markers for extra-pelvic endometriosis.

To date the significance of immunohistochemical staining of TE has not been evaluated. The current review attempts to fill in this gap by comparing histological with immunohistochemical characterization of TE. Furthermore, because pathogenesis of TE is currently controversial, we will also provide some insights into this debate.

### 2.5. Pathogenesis of TE

The cause of thoracic endometriosis is controversially discussed comparable to pelvic endometriosis, although many authors agree that the implantation hypothesis by Sampson [3] is the most accepted. Other theories put forward to explain TE include; coelomic metaplasia, stem cells, lymphatic or hematogenous spread, and the prostaglandin theory [22,32].

## 3. Materials and Methods

We searched in PubMed for articles describing an association between CP and endometriosis (Figure S1). We performed a systematic retrospective literature review starting with 1998, when the first manuscript with immunohistochemical stainings of CP endometrial implants was published [26]. This study presents a review of manuscripts published in several journals between the years 1998 until 2019. We looked for the keywords: thoracic endometriosis syndrome, lung endometriosis, pleural endometriosis, diaphragmatic endometriosis, pneumothorax, catamenial pneumothorax, and all together with endometriosis. Only manuscripts classifying the endometrial implants into glands/stroma, glands, or stroma were included, while those without a clear classification were excluded. We separated the manuscripts into two groups: Group 1. CPE: CP together with immunohistochemistry, which we compared to group 2, CPH: CP together with histology. In both groups the use of histology or immunohistochemistry had to be mentioned at least somewhere in the manuscript.

In both groups the following parameters were also summarized, where possible: age, pain symptoms, imaging techniques, side of implants, markers used, concomitant endometriosis, and recurrence. Values were summarized as means ± standard deviation (SD). Non-parametric comparisons between two groups were done with the Mann–Whitney test. Furthermore, 2 × 2 contingency tables were analyzed with Fisher’s exact test. Analysis was performed with GraphPad InStat3 (GraphPad).

## 4. Results

In total 41 manuscripts (CPE, *n* = 26; CPH, *n* = 15) were reviewed in the present study (Table S1). The mean age of the patients diagnosed with CPE and CPH was 36.3 ± 8.7 and 37.0 ± 6.2, respectively (Table 1 and Table S1). The predominant presentation of CPE and CPH was chest pain (79.1% vs. 82.1%) and dyspnea (88.4% vs. 74.4%). However, especially in the case series the symptoms were rarely recorded. Similarly, the main symptoms of pelvic endometriosis like pelvic pain, dysmenorrhea, abdominal pain, and dyspareunia were seldom presented (Table S1). Concomitant endometriosis was found in 42.8% (CPE) and 62.5% (CPH). Diagnosis and surgery of CPE and CPH was performed predominantly with CTX, CT, and video assisted thoracic surgery (VATS) with 60.9% vs. 37.5%, 36.4% vs. 25.5%, and 75.5% vs. 84.8%, respectively. However, use of MRI was negligible (Table S1).

A very high percentage of right-sided preference of 94.1% (CPE) compared to 89.6% (CPH) was observed. High values were also obtained for diaphragmatic endometrial implants: CPE (96.4%) with 43.4% fenestrations and 32.4% spots (or blebs, plaques, and nodules). Similarly, diaphragmatic endometrial implants in CPH were observed in 83.3% with 21.4% fenestrations and 38.1% spots (Table 1). Spots, blebs, plaques, and nodules were put into one group, because the differences were not clearly defined.

Evaluation of the endometrial implants by immunohistochemistry was most often done with antibodies specific for ER, PR, and CD10 and combinations of them (Table 1 and Table S1). Of note, stromal endometriosis in CPE was identified in 52.7% compared to only 10.2% in CPH, which was highly significant (Table 1). Appearance of stroma or glands/stroma was not correlated with recurrence (Table 2), although recurrence was not reported in all cases.

## 5. Discussion

By and large, the results of this study confirm previous findings showing the high incidence of chest pain, dyspnea, the right-sided preference, the mean age, and the diaphragmatic implants as summarized recently [15]. To avoid redundancy, only relevant and new results are discussed in detail. It was particularly striking that occurrence (or not) of pelvic endometriosis was not mentioned in all studies (Table 1 and Table S1). Similarly, only two manuscripts in the CPE group presented VAS scores for pelvic endometriosis [33,34]. Furthermore, it would have been also interesting to indicate more often smoking habits and pneumothorax recurrence (Table S1). Due to missing data the current review has some limitations, however, several conclusions can be drawn.

The most noteworthy finding of the current review is the very high percentage of stromal endometriosis in the immunohistochemical studies (52.7%) compared to histological investigations (10.2%). Remarkably, in a large study of pelvic endometriosis, also a high percentage of 44.9% (123/274) cases were classified as stromal endometriosis [35]. Stromal endometriosis presents usually in the form of superficial nodules or plaques and is a common form of pelvic endometriosis [35]. Most often CD10 is used for immunohistochemical detection and classification of endometrial stromal cells in TE and in pelvic endometriosis.

CD10 also known as neutral endopeptidase (NEP), common acute lymphoblastic antigen (CALLA), membrane metallo-endopeptidase (MME) or neprilysin in the human endometrium was first described in 1992 [36]. CD10 is expressed throughout the menstrual cycle but absent or only faintly found in decidual stromal cells [36]. Furthermore CD10-positive endometrial stromal cells are also common in adenomyosis and endometriosis [36]. Sensitivity of CD10 in pelvic endometriosis was determined to be 88% (22/25) [37], or 95.83% (46/48) [38] or 85% (17/20) [39] in endometriosis cases, but all sections from lesions that may simulate endometriosis like for example endosalpingiosis were CD10 negative [39]. Although stromal endometriosis was described already in 1960 [40], it did not attract considerable attention over the past decades, mainly because of the general opinion that it is scarce and only the presence of ectopic endometrial glands together with stroma was regarded as a definitive proof of endometriosis [1]. Recently, this view has been challenged and the importance of fibrosis in endometriosis, without ectopic endometrial glands/stroma, was put into the limelight [2]. Although many cell types might be involved in fibrosis such as platelets, macrophages, ectopic endometrial cells, and sensory nerve fibers, the major component of nodular lesions is not endometrial tissue but fibromuscular tissue [41]. However, as long as there is no specific tissue marker with a high specificity available to judge “endometrial fibrosis”, it will be difficult to expand the diagnosis of endometriosis. Similarly, the presence of hemosiderin and/or hemosiderin-laden macrophages, pseudoxanthoma cells or pigmented histiocytes in endometriotic lesions is not conclusive for the diagnosis [1]. In this review, we identified several manuscripts in which only hemosiderin but not glands or stroma was found in lesions of some cases (Table S1), e.g., [33]. Nonetheless, as clearly shown in this review, stromal endometriosis should become an essential part of endometriosis diagnosis. Of note, fibrosis and scarring without the presence of endometriosis was the most frequent difference between laparoscopic and histologic diagnosis of endometriosis [42].

In most manuscripts dealing with immunohistochemistry in TE, antibodies to detect CD10 were mostly used, and often combined with antibodies to detect ER and/or PR. Although CD10 is normally sufficient for detection of endometriosis, the coexpression of CD10 and ER/PR might be helpful in CP diagnosis [29]. Recently, another stromal marker, IFITM1, was evaluated in pelvic and extra-pelvic endometriosis and demonstrated a higher sensitivity (96.8% vs. 100% in 62 cases, respectively) compared to CD10 [28]. Additionally, for the detection of ectopic endometrial epithelial cells PAX8 demonstrated sensitivities of 100% (8/8) in ovarian endometriosis and of 97.9% (46/47) in extragenital epithelial endometrial cells [31]. Of note, Forkhead box L2 (FoxL2), which is expressed in stromal and epithelial endometrial cells, was approximately 3-fold higher expressed in ovarian endometriosis and eutopic endometrium of cases with endometriosis compared to healthy controls, however, no data about the sensitivity was reported [43].

In most manuscripts classification of endometrial implants into glands/stroma, glands and stromal endometriosis was provided, however, sometimes no endometrial implants could be identified in CPE (6.8%) and CPH (30.6%) (Table 1). Similarly, sometimes endometrial implants are not found at the first, but only in subsequent VATS. This might possibly be due to small lesions, missed at the time of the first operation as observed similarly in pelvic endometriosis [44] or atypical lesions, which are not uncommon [42].

There is an urgent need for a better, more sensitive and easier method for detection of endometriosis in patients during surgery. Several methods have been tried such as 5-aminolevulinic acid-induced fluorescence, indigo carmine, methylene blue, indocyanine green, and peritoneal fluid painting, however, up to date, no labeling is currently consistently used [45].

Similarly, thoracic lesions or nodules that are small or more distant from the pleural surface might not be easily detected by palpation or with endoscopic forceps [46]. Thus, preoperative localization of small pulmonary lesions with either metallic materials (e.g., hook wire), with dyes (e.g., methylene blue), with contrast agents (e.g., lipidiol), or radiotracers (technetium-88m) have shown a success rate of 90–100% [46]. These techniques resulted in higher successful VATS rates, shorter operation times, increased economic benefits [46], and possibly longer recurrence-free survival of superficial premalignant, minimally invasive, and small invasive lung cancers [47]. However, the optimal preoperative localization method with respect to success, safety, and efficacy has not yet been established. Although many issues have to be solved, preoperative and operative localization methods for detection of endometrial implants in the thorax might be worth being investigated in the future.

**Figure 1:**
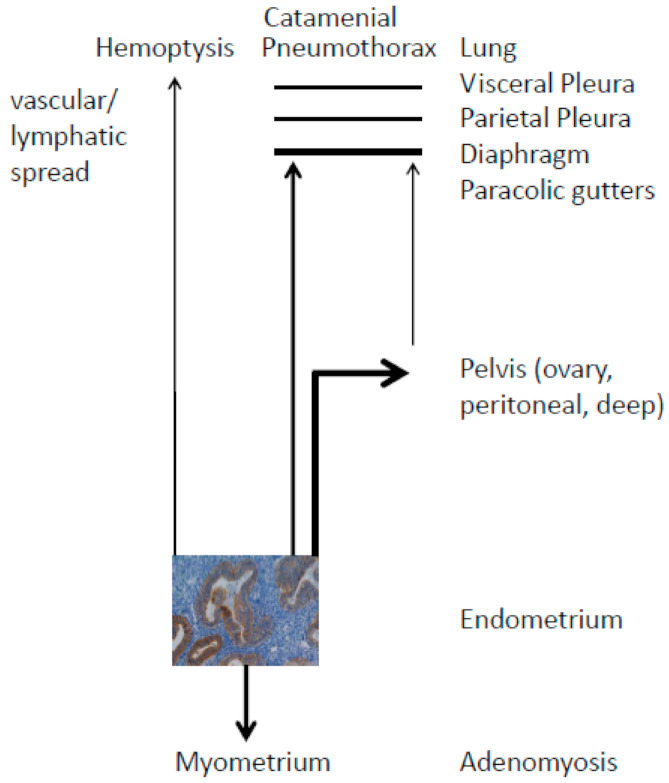
Scheme of the pelvic and thoracic endometriosis. Endometrial glands with the surrounding stroma are the basic unit in the endometrium as shown with claudin-4 positive glands in brown. After tissue breakdown, most often by menstruation, the glands/stroma invade the myometrium, the pelvis, or extra-pelvic sites. The most probable and most common migration of the lands/stroma is via the fallopian tube into the pelvis (retrograde menstruation). However, very rarely the glands/stroma also invade via the right paracolic gutters the right diaphragm, the parietal and visceral pleura and sometimes even the lungs or some other extra-pelvic sites such as for example liver and brain (not shown). Very rarely, bilateral and left-sided invasions in the case of catamenial pneumothorax have been observed. In the case of hemoptysis, the vascular spread of the glands/stroma seems to be preferred. The different thickness of the arrows reflects the relative frequency of endometriosis at different ectopic sites. Modified from Omwandho et al. [54].

Several hypotheses have been postulated to explain the occurrence of thoracic endometriosis, which are all highly similar to the theories of pelvic endometriosis:

1. The coelomic metaplasia theory suggests that endometriosis arises by metaplasia of coelomic epithelial cells. However, this theory cannot explain why the majority of thoracic endometriosis occurs on the right side of the lung in females. Additionally, TE has never been observed in male pneumothorax [48]. Furthermore, no one has up to date demonstrated how one cell type, the coelomic epithelial cell, can differentiate into two distinct cell types, endometrial epithelial and stromal cells, which must happen in an always highly identical manner at many different sites in the body as diverse as the pelvis up to the brain [49]. Recently, we have casted some doubts on the metaplasia hypothesis [49], but as of yet no one has presented convincing data showing metaplasia of peritoneal cells into endometrial epithelial and stromal cells neither in vivo nor in vitro;
2. The stem/progenitor cell theory is mainly advocated by the groups of Gargett [6] and Taylor [50]. They suggest that stem/progenitor cells from the endometrium or bone marrow are responsible for the formation of ectopic endometrial implants. Firstly, the terminology of the cells is somehow diffuse; in the case of pluripotent stem cells, we again have the same problem as with the metaplasia hypothesis, the stem cell must differentiate into two distinct cell types, endometrial epithelial and stromal cells, and this must happen in an identical manner at many different sites in the body [49]. Although a model was presented recently [51], however, no transformation of stem cells into endometrial epithelial and stromal cells has ever been shown to occur at the sites of ectopic endometrial implants. Secondly, in the case of progenitor cells, these cells are already committed to the endometrial lineage, and thus endometrial stromal and epithelial cells can be distinguished. This is not in contradiction to the hypothesis of Sampson, but only an extension. Recently, it was suggested that stem cells might also be the cause of TE [32] and one of the arguments in favor of stem cells was the difference in histologic features between eutopic endometrial tissue and ectopic implants. However, besides the fact that ectopic endometrial implants nearly always have an overtly endometrial phenotype [1], we have recently shown that ectopic endometrial implants are highly similar to eutopic endometrium and have not lost their distinct epithelial characteristics [52].
3. The hypothesis of retrograde menstruation is the most probable theory to explain the phenomenon of pelvic and thoracic endometriosis. The implantation hypothesis of Sampson [3] is based upon: (a) endometrial tissue breakdown, primarily by menstruation, and (b) migration of endometrial cells through either the fallopian tube and pelvis, primarily by retrograde menstruation, or vascular or lymphatic spread, that results in: (c) invasion and implantation at pelvic or extra-pelvic sites (Figure 1) [53].

In other words, several basic cellular processes, such as tissue breakdown, migration, and escape from the immune system, survival and invasion of the endometrial tissue are pivotal for the establishment of endometrial implants [54], although there is limited knowledge about the details and causes of these processes. The whole process leading to endometriosis has many parallels to the seed and soil hypothesis for tumor cells of Paget [55], who proposed that the organ-preference patterns of tumor metastasis are the product of favorable interactions between metastatic tumor cells (the “seed”) and their organ microenvironment (the “soil”). However, it is important to stress several important differences of endometrial to tumor cells: tissue breakdown by the endometrial cells is not necessary, because of menstruation and endometrial cells do not undergo a malignant transformation but only partial transitions [56].

Then, how do the endometrial cells reach the lungs? The endometrial cells follow the clockwise flow of the peritoneal fluid through the right paracolic gutters to reach the right subdiaphragmatic area (Figure 1) [22]. In contrast, the phrenicocolic ligament and the falciform ligament of the liver usually prevent the invasion of the left diaphragm and pleura. Indeed, ectopic endometrial implants have been found at the paracolic gutters [42,57], which is a strong argument in favor of the migration hypothesis, although adhesions at the paracolic gutters are very rare. In contrast, in cases of hemoptysis, the endometrial cells might reach the lungs through hematogenic/lymphatic spread, which was already mentioned by Sampson as recently quoted in a reappraisal of his work [53]. As already shown in 1940, injection of endometrial tissue into the ear vein of rabbits resulted in pulmonary endometriosis in 79% (19/24) of the animals [58]. Similarly, Samani et al. [59] injected labeled endometrial cells into the pelvis, and observed (micro)-metastases in the lungs in vivo. Furthermore, circulating endometrial cells have been found in the blood of patients with spontaneous pneumothorax [60].

Treatment of hemoptysis very often results in complete remission or partial response in all patients treated with hormonal or conservative treatment, which is in strong contrast to the high recurrence rate observed for CP/TE [22]. This does not only suggest that they are distinct entities [22], but one has also to keep in mind that the hematogenous/lymphatic spread of endometrial cells is less frequent compared to retrograde menstruation through the fallopian tube. The recurrence rate does not depend on the rates of diaphragmatic implants with glands/stroma [16] as shown in the present review. Although operation techniques together with a hormonal therapy [61] and a complete resection of all endometrial implants will reduce the recurrence rates, Shakiba et al. [13] showed that patients with pelvic endometriosis treated with hysterectomy combined with laparoscopy experienced a dramatically lower recurrence rate of 96.0%, 91.7%, and 91.7% compared to laparoscopy alone with a recurrence rate of 79.4%, 53.3%, and 44.6% after 2, 5, and 7 years, respectively. These data clearly suggest that especially hysterectomy is associated with a low reoperation rate and that the endometrium is the main source for endometrial cells, although this does not exclude other hypotheses for pathogenesis of endometriosis as mentioned above. Furthermore, data showing recurrence after hysterectomy, alone or in combination with laparoscopy or VATS, are not a counter-argument to Sampson, but should rather further motivate researchers and clinicians to find more sensitive and effective methods to detect endometrial implants during and after surgery. If we apply the razor of Ockham, the implantation hypothesis of Sampson is the simplest theory and thus the more probable compared to the hypothesis of metaplasia and stem cells, which both need more assumptions.

## 6. Conclusions

In summary, the origin of thoracic endometriosis appears to be almost exclusively in the endometrium as already described for pelvic endometriosis although not all causative factors contributing to the initiation and progression of the disease are known. We suggest that the diagnosis of stromal endometriosis with or without immunohistochemical confirmation should become mandatory for pelvic and thoracic endometriosis, and possibly for other extra-pelvic sites. Furthermore, there is an urgent need for more sensitive and effective detection methods to assess endometrial implants during surgery in the pelvis and in extra-pelvic sites. Especially detection of ectopic endometrial implants at extra-pelvic sites should always be accompanied by a careful examination of the pelvis.

## Figures and Tables

**Table 1:** Characteristics of patients with immunohistochemical compared to histological detection.

Parameters	CPE	CPH	*p* Values
Age	36.3 ± 8.7	37.0 ± 6.2	n.s.
Chest pain	82.9%	82.1%	n.s.
Dyspnea	92.7%	74.4%	n.s.
Cough	58.5%	48.7%	n.s.
Imaging			
Chest, X-ray	20.2%	26.5%	n.c.
CT	7.7%	46.9%	n.c.
Operation			
VATS	71.9%	76.0%	n.s.
Lesions found			
Diaphragm	81.8%	85.7%	n.s.
Pleura	31.4%	22.0%	n.s.
Lung	10.5%	8.7%	n.s.
CP, right-sided	90.7%	89.8%	n.s.
CP, bilateral	2.8%	8.2%	n.c.
CP, left-sided	3.7%	0%	n.c.
Concomitant pelvic endometriosis	43.1%	70.7%	0.0012
TE			
Stromal	52.7%	10.2%	<0.0001
Glands/stroma	36.8%	55.1%	=0.0185
Glands	1.4%	0%	n.c.
Positive, n.sp.	2.3%	4.1%	n.c.
Negative	6.8%	30.6%	n.c.
Detection			
ER	95.1%		
PR	94.7%		
CD10	91.3%		
All three	88.3%		

Age is given as means ± standard deviation; CPE, catamenial pneumothorax with immunohistochemistry; CPH, catamenial pneumothorax with histology; TE, thoracic endometriosis; ER, estrogen receptor, PR, progesterone receptor; n.s., not significant; n.sp., not specified; n.c., not calculated because of too many missing or too few values.

**Table 2:** Recurrence of catamenial pneumothorax (CP) cases with glands/stroma or stroma for CPE and CPH.

	Recurrence	No Recurrence	*p* Values
Glands/stroma	10	20	n.s.
Stroma	9	8	n.s.

n.s., not significant; CP, catamenial pneumothorax.

## Data Availability

The data presented in this study are available on request from the corresponding author.

## Supplementary Materials

The following are available online at https://www.mdpi.com/2073-4409/10/1/180/s1, Figure S1: PRISMA flowchart of literature search and data selection, Table S1. References [62,63,64,65,66,67,68,69,70,71,72,73,74,75,76,77,78,79,80,81,82,83,84,85,86,87,88,89,90,91,92,93,94,95,96] are cited in the supplementary materials.
