# Supplementary material for: The Importance of Stromal Endometriosis in Thoracic Endometriosis
Source: Cells. 2021 Jan 18;10(1):180. doi: 10.3390/cells10010180 (PMC7831500; doi:10.3390/cells10010180)
Supplement: Supplementary file 1 [file cells-10-00180-s001.zip › cells-1059106 supp Figure S1.pdf]

cells

MDPI

Supplementary

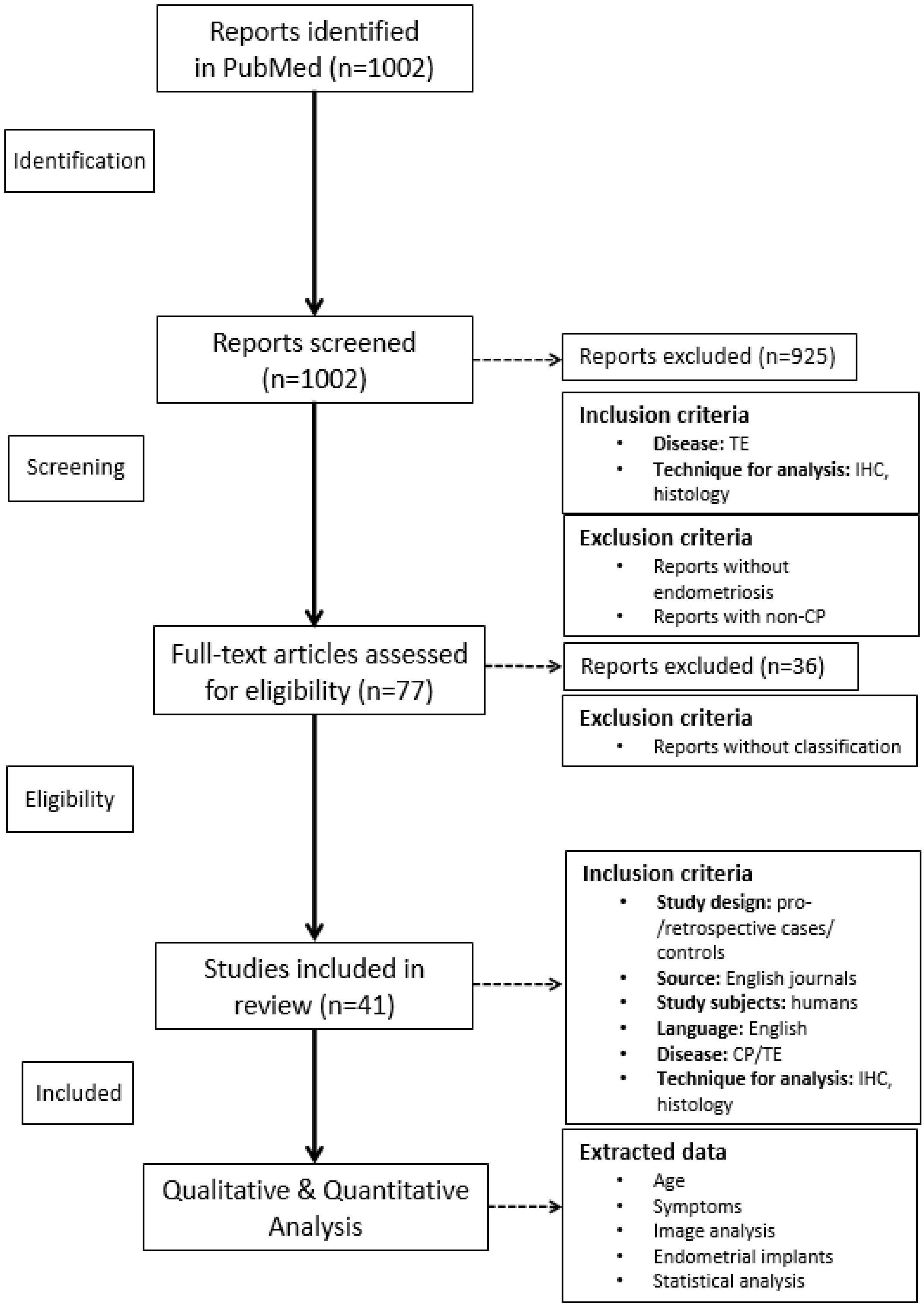

Figure S1. PRISMA flowchart of literature search and data selection. This systematic retrospective review is based upon literature research conducted in PubMed. The main focus was on thoracic endometriosis (TE) with a special focus on catamenial pneumothorax (CP). Only reports containing immunohistochemical (IHC) and histological classification of endometrial implants have been selected in the end. These reports were carefully read and data extracted. All data including missing data have been deduced from the manuscripts and are summarized in Tables 1 and S1 [62–96].
